# 2′-hydroxyl modification improves enzymatic and thermal stability of mRNA

**DOI:** 10.1016/j.omtn.2025.102821

**Published:** 2026-01-14

**Authors:** Satoshi Uchida

**Affiliations:** 1 Department of Advanced Nanomedical Engineering, Medical Research Laboratory, Institute of Integrated Research, Institute of Science Tokyo, 1-5-45 Yushima, Bunkyo-ku, Tokyo 113-8510, Japan; 2 Innovation Center of NanoMedicine (iCONM), Kawasaki Institute of Industrial Promotion, 3-25-14 Tonomachi, Kawasaki-ku, Kawasaki 210-0821, Japan; 3 Pandemic Preparedness, Infection and Advanced Research Center (UTOPIA), The University of Tokyo, 4-6-1, Shirokanedai, Minato-ku, Tokyo 108-0071, Japan

In a recent issue of *Molecular Therapy Nucleic Acids*, Yamada et al. reported that chemical modification of the 2′-hydroxyl groups of mRNA ribose improves enzymatic and thermal stability with reduced but still detectable translational activity.^1^ The 2′-hydroxyl groups are key contributors to mRNA degradation, both through ribonuclease (RNase) attack in physiological environments and hydrolysis during storage. Therefore, chemical modification of the 2′-hydroxyl groups is expected to mitigate both degradation mechanisms. In this study, the authors successfully prepared 2′-hydroxyl-modified mRNA via *in vitro* transcription (IVT) using mutant RNA polymerases, enabling the first comprehensive analysis of the impact of 2′-hydroxyl modification on mRNA stability and translational activity. The findings highlight the potential of 2′-hydroxyl modification as a strategy to address the inherent instability of mRNA in biomedical applications.

mRNA has proven effective in vaccines by encoding antigens and has shown promise in diverse medical applications by encoding therapeutic proteins.^2^ However, its instability, particularly susceptibility to enzymatic degradation and hydrolysis, remains a major challenge.^3^^,^^4^ After *in vivo* administration, mRNA is subject to RNase attack, limiting delivery to target cells. Degradation can occur even after encapsulation in synthetic nanoparticles.^4^ In addition, mRNA undergoes hydrolysis, necessitating stringent low-temperature storage conditions.^4^ The 2′-hydroxyl groups of ribose, a hallmark distinguishing RNA from DNA, are central to both degradation mechanisms. Substitution of the 2′-hydroxyl groups is therefore a promising strategy to enhance RNA stability, as demonstrated in oligonucleotide therapeutics such as small interfering RNA (siRNA).^5^

Applying this strategy to mRNA, however, faces two major challenges: compatibility with transcription and translation. Therapeutic mRNA is typically prepared by IVT using RNA polymerase and a DNA template. Standard RNA polymerases are generally unable to incorporate 2′-hydroxyl-modified nucleotides into the mRNA strand, hindering IVT.^6^ Furthermore, modified mRNA must remain translatable in target cells. Notably, currently approved mRNA vaccines and clinical candidates often employ N1-methyl-pseudouridine modifications, which are relatively well tolerated for transcription and translation. In contrast to such nucleobase modifications, the effects of 2′-hydroxyl modification on these processes remain poorly understood.

To address the transcription challenge, the study employed mutant RNA polymerases previously reported to incorporate modified nucleotides.^6^ To enhance mRNA stability, the authors selected 2′-O-methyl (2′-O-Me) and 2′-fluoro (2′-F) modifications, both well established in siRNA therapeutics. These modifications, used in approved siRNA therapeutics, confer high nuclease stability, enabling administration of naked siRNA without synthetic carriers.^5^ For mRNA IVT, mutant RNA polymerases optimized individually for each modification were employed. These mutants successfully incorporated 2′-O-Me- or 2′-F-modified nucleotides into mRNA across all four base types, although the mutant for 2′-O-Me modification did not accept unmodified nucleotides for two base types (Figure 1A). Based on these results, the authors prepared mRNA with 100% nucleotide substitution by 2′-O-Me-modified nucleotides for two to four base types, and mRNA with 20%–80% substitution by 2′-F-modified nucleotides for guanine or all four bases for subsequent stability and translation analyses (Figure 1B).

**Figure 1:**
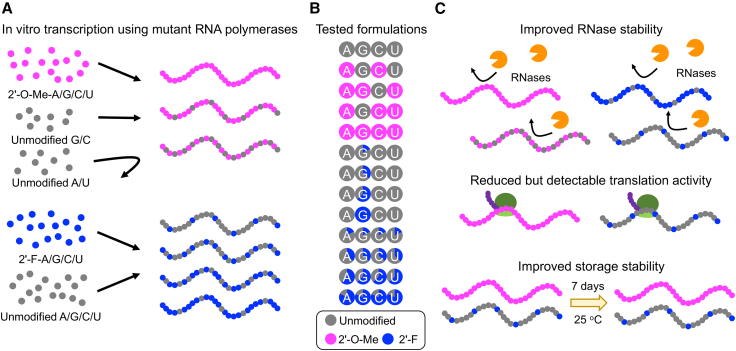
2′-Hydroxyl modification of mRNA (A) Mutant RNA polymerases enabled the incorporation of 2′-modified nucleotides into mRNA via IVT. (B) mRNAs containing different types and percentages of 2′-modified nucleotides were prepared for screening. (C) Optimal mRNA formulations exhibited enhanced stability against RNases and hydrolysis, while maintaining reduced but detectable translational activity.

Nuclease stability was assessed using RNase T1 and RNase A, which have distinct base preferences. Both 2′-O-Me and 2′-F modifications enhanced enzymatic stability when applied to the preferred bases of each RNase. Modification across all four base types effectively suppressed mRNA degradation by both RNases. The protective capability was strengthened by increasing the substitution percentages of modified nucleotides. Moreover, both 2′-O-Me and 2′-F modifications prevented enzymatic degradation in fetal bovine serum, demonstrating their stabilizing effects in a physiological environment.

Translational activity was then evaluated. Reporter protein expression from 2′-O-Me-modified mRNA was reduced by two orders of magnitude compared to unmodified mRNA but remained detectable in cultured cells regardless of modification pattern. This is consistent with the known inhibitory effect of endogenous 2′-O-Me modification on translation.^7^ Similarly, 2′-F modifications reduced translational activity in proportion to substitution percentage. Both 2′-O-Me- and 2′-F-modified mRNAs retained translational activity *in vivo* following intramuscular injection in mice. Notably, mRNA with 20% substitution by 2′-F-modified nucleotides in all four base types exhibited translational activity nearly comparable to unmodified mRNA. Moreover, the modification extended the duration of protein expression, possibly by reducing intracellular mRNA degradation.

Thermal stability was evaluated after 1 week of storage at 25°C. Capillary electrophoresis revealed that 2′-O-Me and 2′-F modifications mitigated degradation compared to unmodified mRNA. Reporter protein expression assays confirmed that modified mRNAs preserved translational activity more effectively after storage, demonstrating enhanced thermostability.

Collectively, this study demonstrates that 2′-hydroxyl modification stabilizes mRNA against RNase degradation and hydrolysis, while maintaining detectable translational activity in cultured cells and mice (Figure 1C). This approach may help address the enzymatic and thermal stability issues that limit current mRNA vaccines and therapeutics. Although nanoparticle encapsulation effectively prevents RNase degradation, it often introduces safety concerns. By contrast, 2′-hydroxyl modification could enable carrier-free delivery systems with improved safety, as observed in siRNA therapeutics.^5^ While systemic delivery of naked mRNA remains challenging, several studies have shown its utility in local delivery to the lungs or skin of rodents and large animals.^8^^,^^9^ The modification could further enhance such local applications of naked mRNA. It could also complement existing delivery systems that struggle with RNase stability.^4^ For thermal stability, current solutions are limited, underscoring the need for new approaches such as 2′-hydroxyl modification.

Nevertheless, the issue of reduced translational activity must be addressed. Potential solutions include exploring alternative 2′-hydroxyl modifications and optimizing the type and percentage of modified nucleotides to balance stability and translation. Another promising strategy is position-specific modification. A recent study demonstrated that 2′-F modification of the first nucleotides in codons improved RNase stability without impairing translation.^10^ However, such position-specific modification cannot be achieved via IVT, which introduces modifications randomly. Instead, it requires chemical synthesis of mRNA, which remains technically challenging for long transcripts encoding therapeutic proteins. Despite these hurdles, 2′-hydroxyl modification holds significant potential to transform the field of mRNA vaccines and therapeutics by addressing the fundamental problem of mRNA instability.

## Declaration of interests

S.U. is a chief medical officer of Crafton Biotechnology Co., Ltd.
